# Potent anti-leukemic activity of a specific cyclin-dependent kinase 9 inhibitor in mouse models of chronic lymphocytic leukemia

**DOI:** 10.18632/oncotarget.25293

**Published:** 2018-05-29

**Authors:** Joachim R. Göthert, Roze Imsak, Michael Möllmann, Stefanie Kesper, Maria Göbel, Ulrich Dührsen, Arne Scholz, Ulrich Lücking, Matthias Baumann, Anke Unger, Carsten Schultz-Fademrecht, Bert Klebl, Jan Eickhoff, Axel Choidas, Jan Dürig

**Affiliations:** ^1^ Department of Hematology, West German Cancer Center (WTZ), University Hospital Essen, Essen, Germany; ^2^ Bayer AG, Pharmaceuticals, Drug Discovery, Berlin, Germany; ^3^ Lead Discovery Center GmbH (LDC), Dortmund, Germany

**Keywords:** chronic lymphocytic leukemia, cyclin-dependent kinase 9, MEC-1 cell line, TCL1 transgenic mice, NSG mice

## Abstract

Onset of progression even during therapy with novel drugs remains an issue in chronic lymphocytic leukemia (CLL). Thus, there is ongoing demand for novel agents. Approaches targeting cyclin-dependent kinases (CDK) have reached the clinical trial stage. CDK9 mediating RNA transcriptional elongation is the evolving pivotal CLL CDK inhibitor target. However, more CDK9 selective compounds are desirable. Here, we describe the CDK9 inhibitor LDC526 displaying a low nanomolar biochemical activity against CDK9 and an at least 50-fold selectivity against other CDKs. After demonstrating *in vitro* MEC-1 cell line and primary human CLL cell cytotoxicity we evaluated the LDC526 *in vivo* effect on human CLL cells transplanted into NOD/scid/γc^null^ (NSG) mice. LDC526 administration (75 mg/kg) for 5 days resulted in a 77% reduction of human CLL cells in NSG spleens compared to carrier control treatment. Next, we longitudinally studied the LDC526 impact on circulating CLL cells in the TCL1 transgenic mouse model. LDC526 (50 mg/kg) administration for two days led to a 16-fold reduction of blood CLL cell numbers. Remarkably, residual CLL cells exhibited significantly increased intracellular BCL-2 levels. However, the LDC526 cytotoxic effect was not restricted to CLL cells as also declining numbers of normal B and T lymphocytes were observed in LDC526 treated TCL1 mice. Taken together, our *in vivo* data provide a strong rational for continued LDC526 development in CLL therapy and argue for the combination with BCL-2 inhibitors.

## INTRODUCTION

CLL is the most common leukemia in Western countries [1]. The clinical course of CLL is highly variable. Specific biologic and clinical factors are associated with rapid progression towards more advanced disease stages in some patients whereas others survive for a long period without the need for treatment [2]. Compared to other hematologic malignancies CLL is a nonproliferative leukemia characterized by the over-expression of antiapoptotic BCL-2 family proteins [3]. Survival signals are provided by the highly specialized microenvironment of CLL [4–6]. It is possible to achieve minimal residual disease-negative CLL remissions with combination chemo-immunotherapy in a subset of patients [7], however the majority of patients will inevitably relapse. This will likely also hold true in the current era of novel anti-CLL drug approvals targeting Bruton's tyrosine kinase (BTK), phosphatidylinositol-3-kinase delta (PI3Kδ) and B-cell lymphoma/leukemia 2 (BCL-2) [8]. Patients treated with these novel agents still progress after treatment or may not tolerate them. Treatment failure with the novel agents may occur due to acquired resistance, which in the case of the BTK inhibitor ibrutinib was shown to be mediated by mutations in the ibrutinib-binding site of BTK [9]. Furthermore, patients also progress under treatment with the BCL-2 inhibitor venetoclax. It is assumed that venetoclax resistance is conferred by the overexpression of pro-survival proteins other than BCL-2 such as Myeloid cell leukemia 1 (MCL-1) [10–12]. Likely, *a priori* CLL dependence on MCL-1 rather than BCL-2 [13] conveys decreased venetoclax sensitivity in a subgroup of patients. Additionally, CLL MCL-1 expression is associated with the presence of poor prognostic markers and disease progression [14].

MCL-1 is a protein with a short half-life and its cellular levels are thus susceptible to transient inhibition of RNA transcription [15–17]. RNA transcription and in particular elongation are dependent on cyclin-dependent kinase 9 (CDK9) mediated serine phosphorylation of the RNA Polymerase II (RNAPII) carboxyterminal domain (CTD). CDK9 together with its cyclin partners (T or K) forms a functional complex termed positive transcription elongation factor b (pTEFb). The first generation CDK9 inhibitors such as SNS-032 or Alvocidip (flavopiridol) also targeting other cyclin-dependent kinases are capable of inducing apoptosis of CLL cells [18, 19]. However, the clinical development of these compounds was negatively impacted by their side effect profile in particular by the occurrence of cytopenias, gastrointestinal symptoms and tumor lysis syndrome [20–22]. Likely, the combinatorial inhibition of multiple CDKs contributed to this side effect spectrum. The next-generation CDK inhibitor Dinaciclib specific for CDK1, CDK2, CDK5 and CDK9 was more efficient in inducing CLL apoptosis than flavopiridol [23, 24] and exhibited an improved safety profile [25, 26]. Nonetheless, the occurrence of cytopenias was still reported in Dinaciclib clinical trials [25, 26].

To further increase CDK9 inhibitor specificity and to enable oral administration we developed the novel CDK9 inhibitor LDC526. A recent further pharmacologically optimization of LDC526 resulted in BAY1143572 [27], which has been studied in phase I trials in patients with acute leukemia and solid tumors / lymphomas (ClinicalTrials.gov, Identifier NCT02345382 and NCT01938638, respectively). Here, we report anti-CLL activity of LDC526 in the CLL-derived cell line MEC-1 and in primary CLL cells. Moreover, we demonstrated effective anti-CLL activity of LDC526 in CLL xenografted NSG and TCL1 transgenic CLL mice. In these models LDC526 treatment also decreased non-malignant T cells, which represent an important component of the CLL microenvironment. High BCL-2 expression likely enabled a small fraction of CLL cells to escape LDC526-induced apoptosis.

## RESULTS

### LDC526 inhibits survival of MEC-1 and primary CLL cells

**Figure 1:**
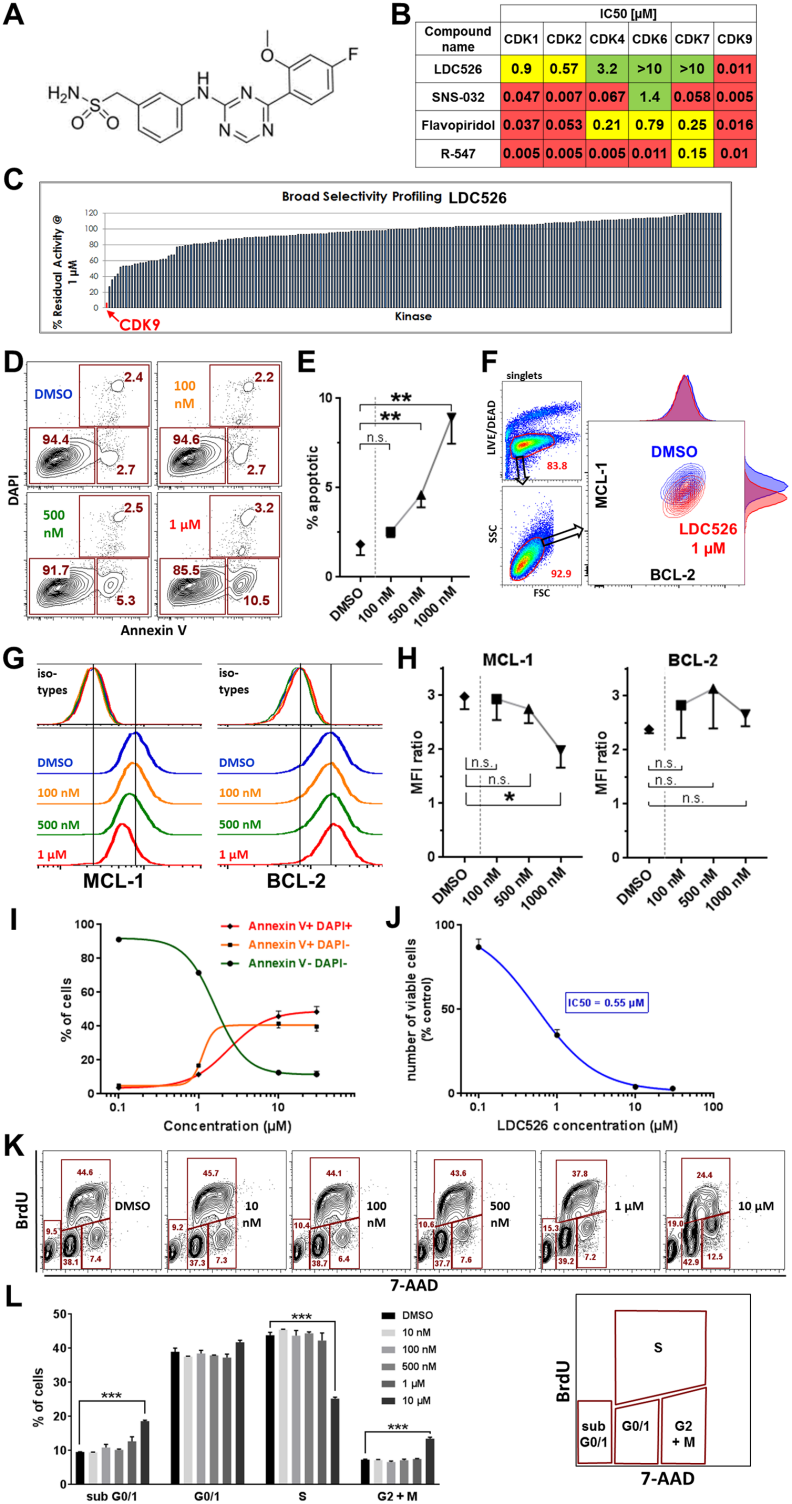
LDC526 is a potent CDK9 inhibitor inducing apoptosis of the MEC-1 cell line **(A)** Molecular structure of LDC526. **(B)** Analysis of CDK family selectivity of LDC526 in comparison to other CDK inhibitors. Red: IC_50_ <0.1 μM; yellow: IC_50_ ≥0.1 and <1μM, green: IC_50_ ≥1 μM. **(C)** High CDK9 specificity of LDC526 in a panel of 219 kinases. **(D)** Rapid induction of apoptosis by LDC526. Apoptosis was assessed by Annexin V and DAPI staining after 4 hours of LDC526 incubation. Representative plots are shown. **(E)** Quantification of apoptotic cells (Annexin V+, DAPI-; n=3 independent replicates; incubation for 4 hours). **(F)** Intracellular flow cytometric analysis of MCL-1 and BCL-2 expression within living (LIVE/DEAD dye negative) MEC-1 cells after 4 hours of LDC526 incubation. Overlay plots (DMSO and LDC626 1 μM) with adjunct histograms are displayed. A representative plot is shown. **(G)** Representative histograms of intracellular MCL-1 and BCL-2 staining (with corresponding isotype controls) of MEC-1 cells after 4 hours of LDC626 incubation. **(H)** Quantification of intracellular MCL-1 and BCL-2 expression (MFI ratio: MFI anti-BCL-2 or anti-MCL-1/MFI corresponding isotype control antibody) after 4 hours of LDC626 incubation (n=3 independent replicates). **(I)** Increasing LDC526 concentrations decreased the proportion of viable (negativity for Annexin V and DAPI staining, determined by flow cytometry) and increased the proportions of apoptotic (Annexin V+, DAPI-) and dead (Annexin V+, DAPI+) MEC-1 cells (n=4 independent wells; 48 hours incubation). **(J)** LDC526 dose-response-curves depicting MEC-1 absolute viable cell numbers (n=4 independent wells; 48 hours incubation). **(K)** Representative plots of MEC-1 cell cycle analysis (flow cytometric BrdU cell cycle assay) after 24 hours of LDC526 incubation. **(L)** Quantification of MEC-1 cell cycle distribution after 24 hours of LDC526 incubation (n=3 independent replicates). ^*^ p<0.05; ^**^ p<0.01; ^***^ p<0.001.

A program for the generation of specific CDK9 inhibitors resulted in the synthesis of the highly selective CDK9 inhibitor LDC526 (Figure 1A). Half-maximal inhibitory doses (IC50) for the CDK kinases 1/2/4/6/7 and 9 were determined. Versus CDK9 LDC526 had a 52/82/291/>900/>900-fold selectivity compared to CDK2/1/4/6/7. In contrast, the other three compounds tested displayed a much lower CDK9 selectivity (e.g.: versus CDK9, Flavopiridol had a 3/2/13/49/16-fold selectivity compared to CDK2/1/4/6/7) (Figure 1B). Next, we performed *in vitro* selectivity kinase profiling with LDC526 using a panel of 219 recombinant kinases. More than 85% of tested kinases still displayed an activity of greater than 80% at a 1 μM concentration of LDC526 (Figure 1C). Taken together, the functional *in vitro* kinase assays demonstrated CDK9 selectivity of LDC526.

**Figure 2:**
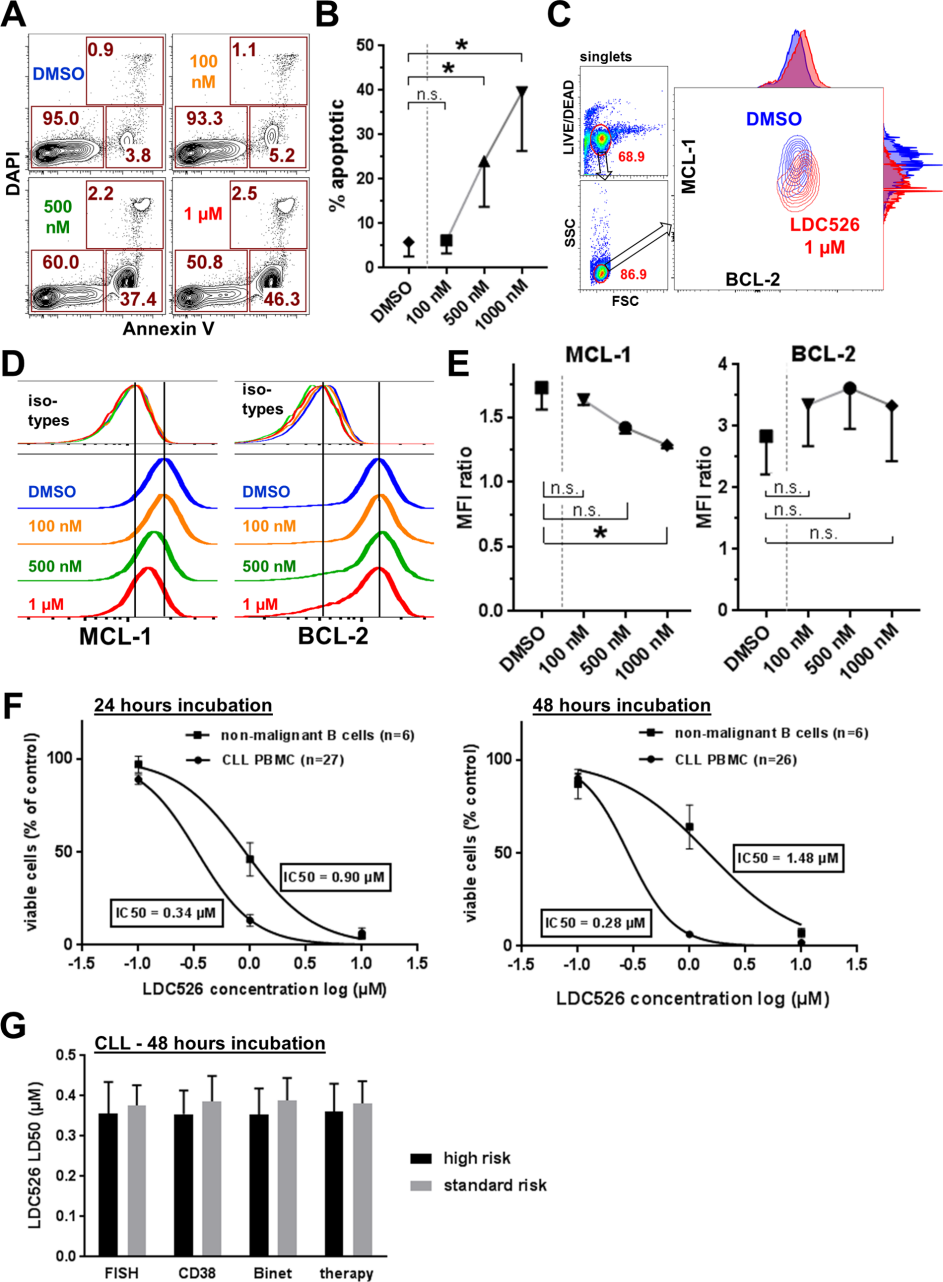
Antileukemic activity of LDC526 on primary CLL cells *in vitro* **(A)** Induction of apoptosis in primary CLL cells by LDC526. Apoptosis was assessed by Annexin V and DAPI staining after 4 hours of LDC526 incubation. Plots of a representative analysis are shown. **(B)** Quantification of apoptotic cells (Annexin V+, DAPI-; n=4 CLL patients; 4 hours of incubation). **(C)** Representative intracellular flow cytometric analysis of MCL-1 and BCL-2 expression within living (LIVE/DEAD dye negative) CLL cells after 4 hours of LDC526 incubation. Overlay plots (DMSO and LDC626 1 μM) with adjunct histograms are displayed. **(D)** Representative histograms of intracellular MCL-1 and BCL-2 staining (with corresponding isotype controls) of primary CLL cells after 4 hours of LDC526 incubation. **(E)** Quantification of intracellular MCL-1 and BCL-2 expression (MFI ratio: MFI anti-BCL-2 or anti-MCL-1/MFI corresponding isotype control antibody) after 4 hours of LDC526 incubation (n=3 CLL patients). **(F)** LDC526 dose-response curves showing the absolute number of viable CLL PBMCs (% of DMSO control) and normal donor B cells determined with absolute cell counts and Annexin V/DAPI flow cytometric analysis. **(G)** LDC526-induced antileukemic effect within different prognostic CLL subgroups. High-risk: FISH, 11q or 17p deletion (n=10); CD38, proportion of CD38+ cells ≥30% (n=14); Binet, Binet B or C (n=12); therapy, previous therapy (n=10). Low-risk: FISH low-risk, (n=18); proportion of CD38+ cells <30%, (n=14); Binet A (n=16); no therapy (n=18). Characteristics of the CLL patient cohort included in this study are outlined in Table 1. Means±SEM are shown, no statistically significant differences between prognostic CLL subgroups (p>0.05) using Mann-Whitney-U test.

As CDK9 inhibition should rapidly decrease levels of the prosurvival MCL-1 protein we studied apoptosis of the CLL-derived cell line MEC-1 after short-term LDC526 exposure (4 hours). Indeed, significantly increased MEC-1 apoptosis was observed at LDC526 concentrations of 500 nM and more pronounced at 1 μM (Figure 1D, 1E). In parallel, we studied intracellular MCL-1 and BCL-2 expression on the single cell level using an intracellular flow cytometric assay (Figure 1F). In contrast to BCL-2, MCL-1 protein levels were reduced in the presence of increasing LDC526 concentrations (Figure 1F, 1G, 1H). Longer-term LDC526 exposure also induced apoptosis (Figure 1I) and decreased the number of viable MEC-1 cells at different concentrations (Figure 1J). To demonstrate that LDC526 primarily induces apoptosis rather than inhibiting proliferation we studied cell cycle progression by BrdU incorporation at different LDC526 concentrations (Figure 1K). In contrast to the impact on cell survival, LDC526 did not alter the cell cycle at concentrations up to 1 μM. Solely at very a high concentration such as 10 μM S-phase entry was significantly decreased (Figure 1L). Next, we studied whether the MEC-1 cell line data were reproducible with primary CLL samples. CLL PBMCs were exposed to LDC526 for four hours and apoptosis was determined with Annexin V staining. Strikingly, significantly increased percentages of apoptosis were observed at concentrations of 500 and 1000 nM (Figure 2A, 2B). Furthermore, increasing LDC526 concentrations led to significantly decreased MCL-1 protein levels while BCL-2 levels were not significantly altered (Figure 2C, 2D, 2E).

Subsequently, we investigated the *in vitro* cytotoxic impact of LDC526 on primary CLL cells (patient characteristics, Table 1) in comparison to healthy donor B cells after longer-term LDC526 exposure (Figure 2F). The number of viable (Annexin V-/DAPI-) CLL cells decreased with increasing LDC526 concentrations. Remarkably, normal donor B cells also displayed sensitivity towards LDC526. However, the IC_50_ of primary CLL cells was lower than the IC_50_ of non-malignant normal donor B cells. This difference in sensitivity was more pronounced at 48 hours than at 24 hours of incubation (Figure 2F). Finally, we analyzed whether prognostic CLL subgroups (Table 1) had a significant impact on LDC526-induced apoptosis. CLL patients were split into subgroups with respect to fluorescent *in situ* hybridization (FISH) genetic aberrations, CD38 surface expression, Binet stage and treatment history. In accordance with the assumption that even high-risk CLLs with e. g. p53 aberrations should be susceptible to the transcriptional elongation inhibition by LDC526 no statistically significant differences were observed between subgroups (Figure 2G). Taken together, LDC526 displayed efficient cytotoxic activity against CLL cells *in vitro*. As expected, LDC526 decreased CLL MCL-1 protein levels and triggered apoptosis rather than inhibiting proliferation.

**Table 1:** Patient characteristics

Parameters	No. of patients (%)
Total number of patients	37 (100)
Mean age 64.6 years (range 43-80)	
female/male	15/22 (41/59)
**Binet stage (n=37)**	
A	23 (62)
B	5 (14)
C	9 (24)
**CD38 expression (n=37)**	
positive	18 (49)
negative	19 (51)
**Genomic aberrations by FISH (n=35)**	
low-risk	24 (69)
high-risk (deletion 11q or 17p)	11 (31)
**Treatment history (n=37)**	
treated	13 (35)
untreated	24 (65)

### LDC526 decreased human CLL numbers in CLL xenografted NSG mice

In addition to the cytotoxic effect on CLL cells, selective CDK9 inhibition is likely to have pleiotropic effects *in vivo*. These include effects on the microenvironment of CLL cells comprising different cell types such as myeloid cells and T cells. Furthermore, proliferating activated CLL cells only exist in specific organ compartments such as lymph nodes, spleen and bone marrow. Therefore, we investigated the impact of specific pharmacologic CDK9 inhibition by LDC526 on CLL *in vivo*.

**Figure 3:**
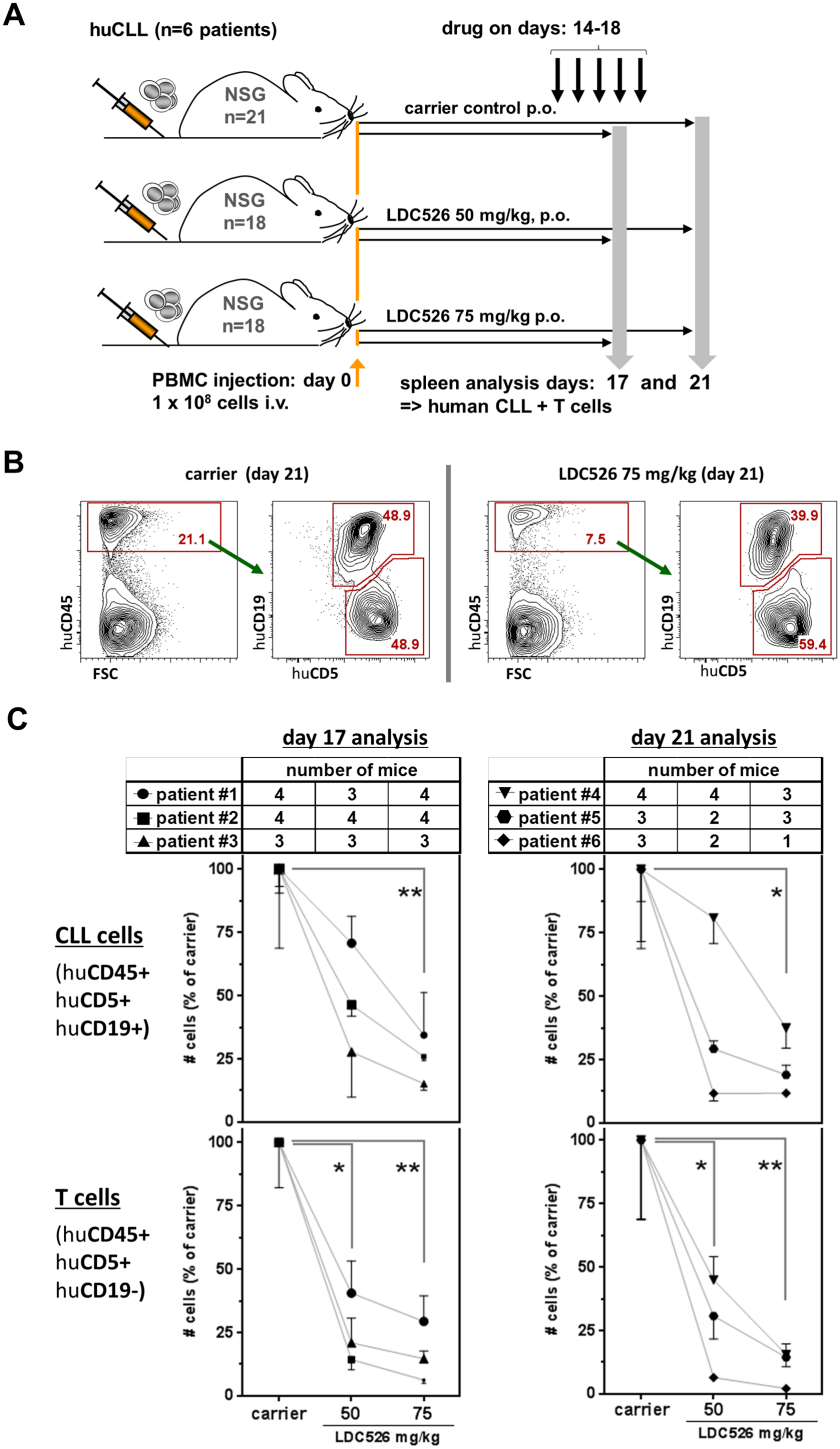
LDC526 decreases splenic human CLL numbers in xenografted NSG mice **(A)** Scheme of the experimental set-up. CLL cells of individual patients were i.v. injected into n=3-4 NSG mice per treatment group (carrier control, LDC526 50 mg/kg and 75 mg/kg). One cohort of NSG mice transplanted with cells from n=3 individual CLL patients was analyzed on day 17. Another cohort of NSG mice transplanted with cells from n=3 further CLL patients was analyzed on day 21. **(B)** Representative flow cytometric analysis (day 21) of splenocytes of human CLL transplanted NSG mice treated with carrier or LDC526, respectively. **(C)** Quantification of splenic human CLL and splenic human T cells after carrier control and LDC526 treatment, respectively. Each data point represents the mean±SEM of NSG spleen cell numbers per individual CLL patient normalized to the mean cell numbers detected in spleens of corresponding carrier control treated mice (transplanted with the same leukemia). Mean CLL cell numbers were statistically compared in paired analyses. Tables depict the numbers of recipient NSG mice analyzed per data point. ^*^ p<0.05; ^**^ p<0.01.

**Figure 4:**
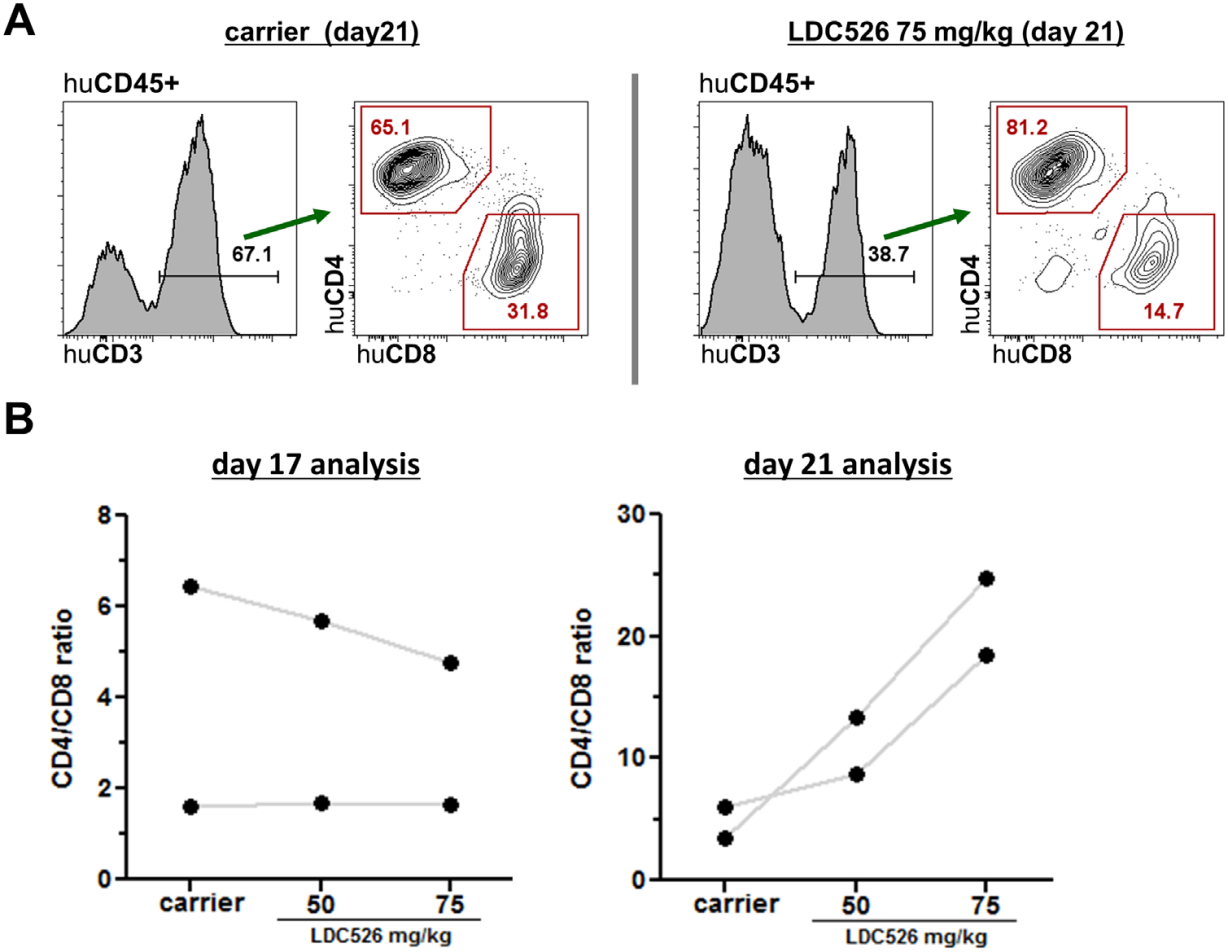
Analysis of human CD4+ and CD8+ T cell subsets in CLL transplanted NSG spleens **(A)** Representative human T cell flow cytometric analysis (day 21) of spleens of human CLL transplanted NSG mice treated with carrier or LDC526 for 5 days, respectively (experimental scheme as outlined in Figure 3A). **(B)** CD4/CD8 ratios of residual human T cells in NSG spleens after LDC526 treatment were determined. Each data point represents the calculated mean CD4/CD8 ratio of residual T cells per individual CLL patient (day 17 analysis: n=2 patients; day 21 analysis: n=2 patients).

We and others previously established human CLL xenograft models using NOD/SCID and NSG mice, respectively [28–30]. We chose to use the NSG model for LDC526 experiments since NSG CLL and concomitant T cell engraftment levels are expected to be higher than in the NOD/SCID model [28–30]. We intravenously injected PBMCs from n=6 CLL patients into n=57 NSG mice and commenced LDC treatment with a 14 day latency according to the experimental set-up outlined in Figure 3A. To study the effect of doses and treatment duration we chose to administer 50 and 75 mg/kg for three days (NSG transplanted with cells originating from n=3 patients) and for five days (NSG transplanted with cells originating from n=3 additional patients). On experimental days 17 and 21 NSG spleens were harvested and the content of human CLL and T cells was determined by cell counting and flow cytometry (Figure 3B). The LDC526 impact on splenic NSG human CLL cell numbers was separately evaluated per originating CLL patient as engraftment was shown to vary between patients [28, 30]. Compared to carrier control treatment, numbers of human CLL cells decreased after 3 and 5 days of LDC526 treatment, respectively. However, statistical significance regarding the reduction of human CLL cell numbers was only achieved at the LDC526 75 mg/kg dose level (Figure 3C). In contrast, the numbers of splenic human T cells were significantly reduced at both LDC526 doses administered (Figure 3C). In order to determine whether there was differential *in vivo* LDC526 sensitivity of T cell subsets we performed additional staining for human CD4+ and CD8+ T cells present in the spleens of xenografted NSG mice (Figure 4A). The human CLL T cell compartment originating from n=2 CLL patients analyzed after 3 days LDC526 treatment exhibited a lower in one case and a constant CD4/CD8 ratio in the other case. In contrast, in mice transplanted with cells of n=2 other CLL patients and receiving LDC526 treatment for 5 days we observed a rising CD4/CD8 ratio with increasing LDC526 doses (Figure 4B). This could mean that CD8+ cytotoxic T cells are more susceptible to longer LDC526 exposure (5 versus 3 days) than CD4+ T cells. However, this differential skew within the T cell compartment could also reflect the heterogeneity of T cells derived from different CLL patients. On days 14-16 of the experimental plan (days 1-3 of LDC526 administration) mice did not show any signs of distress. A mean weight loss of 1.8% was detectable in mice of the LDC526 75 mg/kg group (day 16). However, on the last day of the experiment (day 21) mice of the LDC526 75 mg/kg dose group showed beginning signs of declined general condition with decreased general activity and the mean weight loss was 3.6% (Supplementary Figure 1A). In conclusion, LDC526 displayed significant *in vivo* anti-human CLL activity. Remarkably, human T cell numbers were already decreased at a lower dose level than human CLL cells.

### LDC526 shows significant CLL cell cytotoxicity in TCL1 transgenic mice

**Figure 5:**
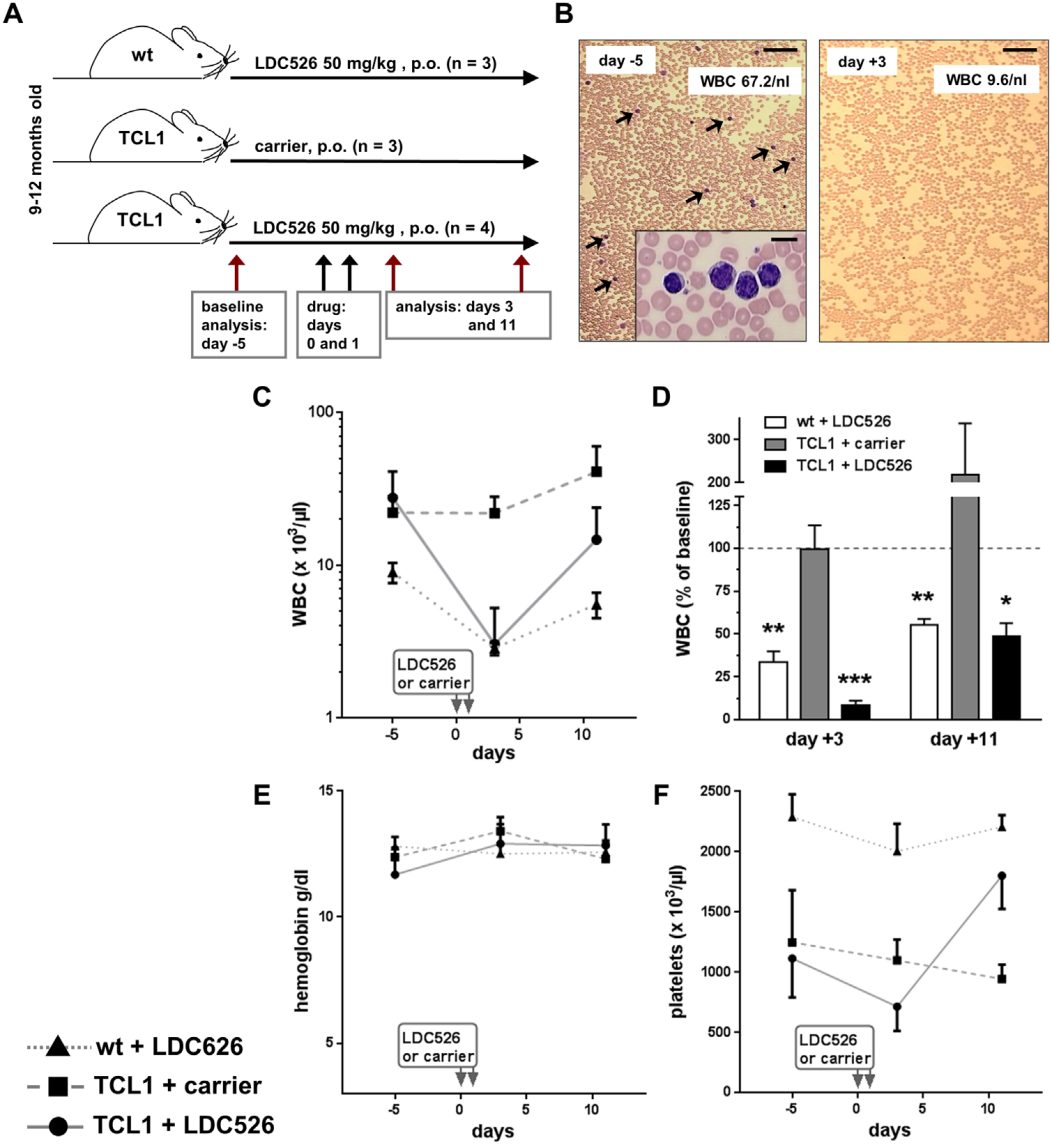
Peripheral blood count analysis of LDC526-treated TCL1 mice **(A)** Scheme of the experimental set-up. **(B)** Sequential peripheral blood smears of a representative LDC526-treated TCL1 mouse. Arrows point at leukemic cells. Inset: Higher magnification of a group of leukemic cells. Images of baseline (day -5) and day +3 smears are shown. Scale bar, 100 μM; inset scale bar 10 μm. **(C)** Longitudinal analysis of white blood cell counts (WBC). **(D)** Percent of WBC relating to baseline values (day -5). Longitudinal analysis of peripheral blood **(E)** hemoglobin and **(F)** platelet concentrations. ^*^ p<0.05; ^**^ p<0.01; ^***^ p<0.001 compared to baseline values. Means+/-SEM are shown.

Another widely used preclinical CLL model are TCL1 transgenic mice [31]. The TCL1 transgenics represent a constitutive CLL model not involving the adoptive transfer of cells. This model mirrors the natural course of human *IgVH*-unmutated CLL including its disease progression [32, 33]. In addition, this model has also been widely used by others to test novel therapeutic molecules [34–36]. Hence, we used TCL1 transgenic mice to investigate the *in vivo* anti-CLL activity of LDC526. Consecutive blood sampling allowed us to carry out a longitudinal study in individual TCL1 and control mice. For this, we chose a cohort of TCL1 and littermate control mice, which were 9-12 months old (Figure 5A). We chose TCL1 transgenics with high peripheral CLL cell (CD19+CD5+) counts (14.6±7.4/nl, mean±SEM). Given the presumably high leukemic burden in these aged TCL1 mice and the reduced general condition of some NSG mice receiving 75 mg/kg for 5 days we decided to dose TCL1 mice with 50 mg/kg daily for two consecutive days (Figure 5A). As the experiment was longitudinally designed and there was no effect expected of administering carrier to wild-type control mice we omitted this control group and established three experimental groups (Figure 5A). In order to minimize stress by collecting blood specimens we sampled blood three times, 8 days apart (days -5, +3 and +11; Figure 5A). Compared to blood smears prepared before commencing LDC526 treatment a marked decrease of peripheral blood leukocytes and CLL cells was observed two days post the last LDC526 administration to TCL1 transgenic mice (Figure 5B). In contrast to the carrier control treated TCL1 mice, white blood cell counts (WBC) dropped in LDC526-treated wild-type and TCL1 mice (Figure 5C). In comparison to wild-type control mice, where WBC dropped to 34% of baseline values, the WBC decrease in TCL1 mice was more dramatic dropping to 8% of baseline values (Figure 5D). While hemoglobin counts did not change significantly (Figure 5E) platelet counts of LDC526-treated mice were decreasing. In contrast to platelet counts of LDC526-treated wild-type mice, TCL1 platelet counts were even rising to significantly higher levels (day +11; p<0.01) than measured at baseline (Figure 5F).

**Figure 6:**
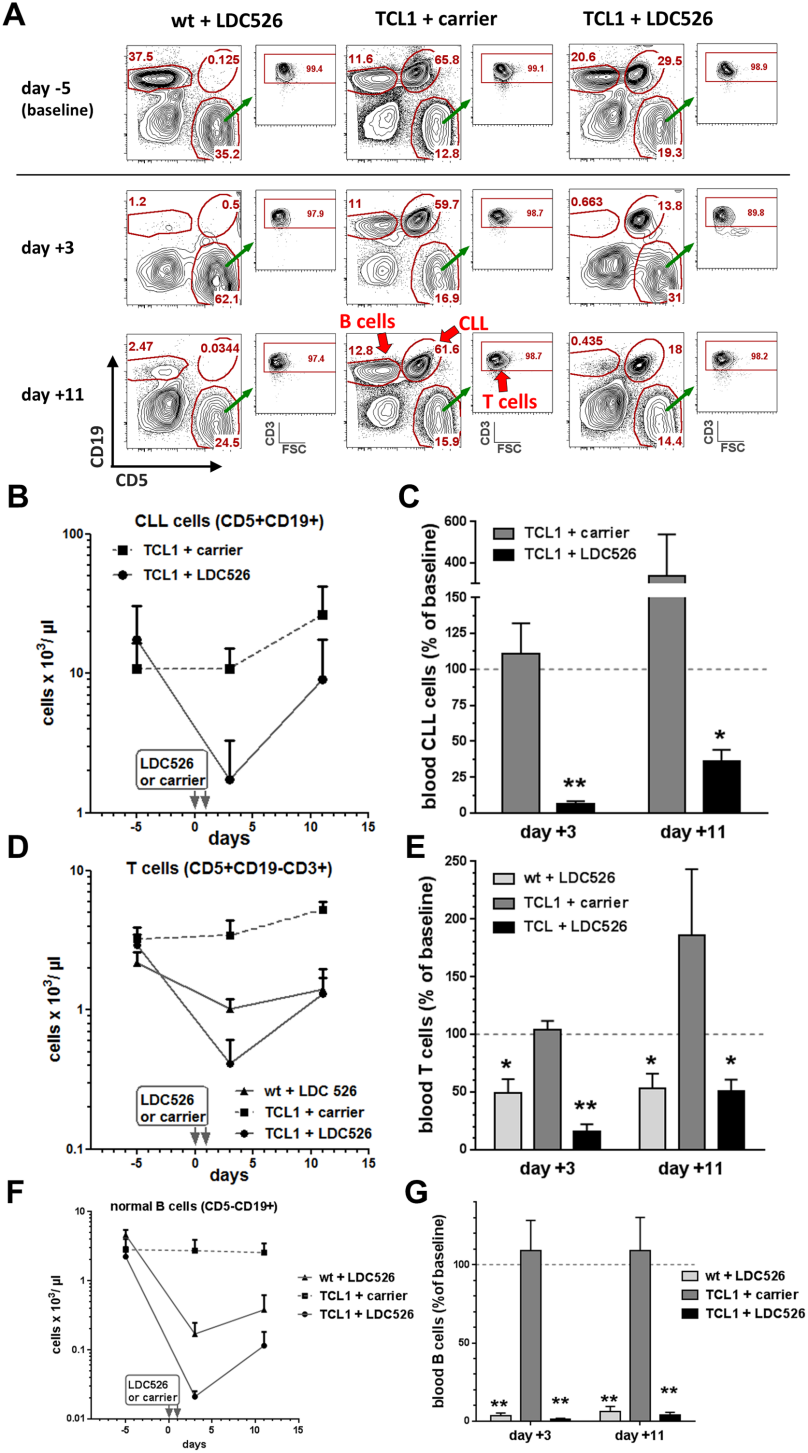
LDC526 treatment depletes peripheral blood CLL cells **(A)** Representative flow cytometry plots of peripheral blood leukocytes (experimental scheme outlined in Figure 5A) stained with antibodies against CD5, CD19 and CD3 defining TCL1 CLL cells (CD5+CD19+), T cells (CD5+CD19-CD3+) and non-malignant B cells (CD5-CD19+), red arrows. By previous gating doublets (FSC-A vs. FSC-W) and dead cells (DAPI+) were excluded from the analysis. **(B)** Longitudinal quantification of circulating CLL cells. **(C)** Percent of CLL numbers relating to baseline values (day -5). **(D)** Longitudinal quantification of circulating T cells. **(E)** Percent of T cell numbers relating to baseline values (day -5). **(F)** Longitudinal quantification of non-malignant blood B cells. **(G)** Percent of non-malignant B cell numbers relating to baseline values (day -5). ^*^ p<0.05; ^**^ p<0.01.

Peripheral blood CLL and non-malignant T and B lymphocyte subsets were defined by flow cytometry using antibodies against CD3, CD5 and CD19 (Figure 6A). Two days after the last LDC526 application TCL1 CLL cell concentrations decreased while CLL concentrations of TCL1 control mice did not change (Figure 6B). Peripheral blood CLL counts of individual LDC526-treated TCL1 mice decreased at least 8-fold (Supplementary Figure 2). Remarkably, mean TCL1 CLL numbers decreased 16-fold compared to baseline levels demonstrating a striking anti-leukemic effect of LDC526 (Figure 6C). In concordance with TCL1 CLL cells, TCL1 blood T cell concentrations also dropped (Figure 6D). Compared to the 16-fold decline of TCL1 CLL cells the decrease of T cells was not as dramatic (6-fold). However, in contrast to TCL1 T cells the concentration of wild-type T cells only halved by LDC526 treatment (Figure 6E).

We also determined the absolute non-malignant B cell numbers within the peripheral blood. While non-malignant B cell numbers of carrier control-treated TCL1 mice remained constant, LDC526 treatment led to a substantial decline of this lymphocyte population (Figure 6F). Non-malignant B cell numbers of LDC526-treated TCL1 mice almost dropped by two log levels to 1.4% of baseline values (Figure 6G). B cells of LDC526-treated wild-type mice also dropped dramatically to 3.7% of baseline levels. As expected, weight loss of LDC526-treated TCL1 mice was more pronounced than the LDC526-induced weight-loss of wild-type mice. All but one LDC526-treated TCL1 mouse, which had to be euthanized due to reduced clinical condition on day +7, regained their weight by day +8 (Supplementary Figure 1B). As the LDC526-associated weight loss was more severe in TCL1 transgenic mice than in wild-type control mice weight loss was at least in part associated with treatment response. In summary, LDC526 displayed an efficient cytotoxic effect against peripheral blood TCL1 CLL cells. Additionally, non-malignant T and B cell numbers also declined after LDC526 treatment.

On day +11 (Figure 5A) the experiment was terminated as planned and spleens of the experimental mice were harvested. To be able to assess the impact of LDC526 on wild-type spleens we included n=3 of non-treated age-matched wild-type littermate mice as an additional experimental control group in the final spleen analysis. A caveat of this final spleen analysis posed the fact that in contrast to the longitudinal blood analysis, baseline spleen data of individual aged TCL1 mice were not available. As longitudinal analysis was not possible, we now compared spleen parameters between cohorts. Importantly, it is unknown to which extent the amount of circulating peripheral blood TCL1 CLL cells determined at baseline correlates with splenomegaly. Nonetheless, TCL1 spleens of mice receiving LDC526 were macroscopically smaller than spleens of carrier-treated TCL1 mice (Supplementary Figure 3A). Consistent with this, splenic weights of LDC-treated TCL mice were significantly lower than the splenic weights of TCL1 mice receiving carrier control treatment suggesting a therapeutic effect (Supplementary Figure 3B). There was also a trend (p=0.09) towards a lower splenic absolute CLL cell content (Supplementary Figure 3C). Here, it has to be taken into account that LDC526 treatment was completed 10 days before the spleen analysis. Thus, as observed in the peripheral blood where CLL numbers increased again by 29% on day +11 compared to day +3 (Figure 6C) also splenic CLL cell numbers might have recovered during this period. In concordance with the peripheral blood data, TCL1 splenic T cells after LDC526 treatment were significantly decreased compared to TCL1 splenic T cells of carrier control treated mice (Supplementary Figure 3D). The LDC526 B cell cytotoxicity observed in the longitudinal blood analysis was also apparent when comparing absolute splenic B cell numbers of wild-type mice with and without LDC526 treatment (Supplementary Figure 3E). Taken together, spleens of LDC526-teated TCL1 mice were smaller than control TCL1 spleens implying a therapeutic benefit. The data also suggest that LDC526 treatment was capable of reducing TCL1 T cell numbers.

### Residual TCL1 CLL cells after LDC526 treatment exhibit high levels of Bcl-2

**Figure 7:**
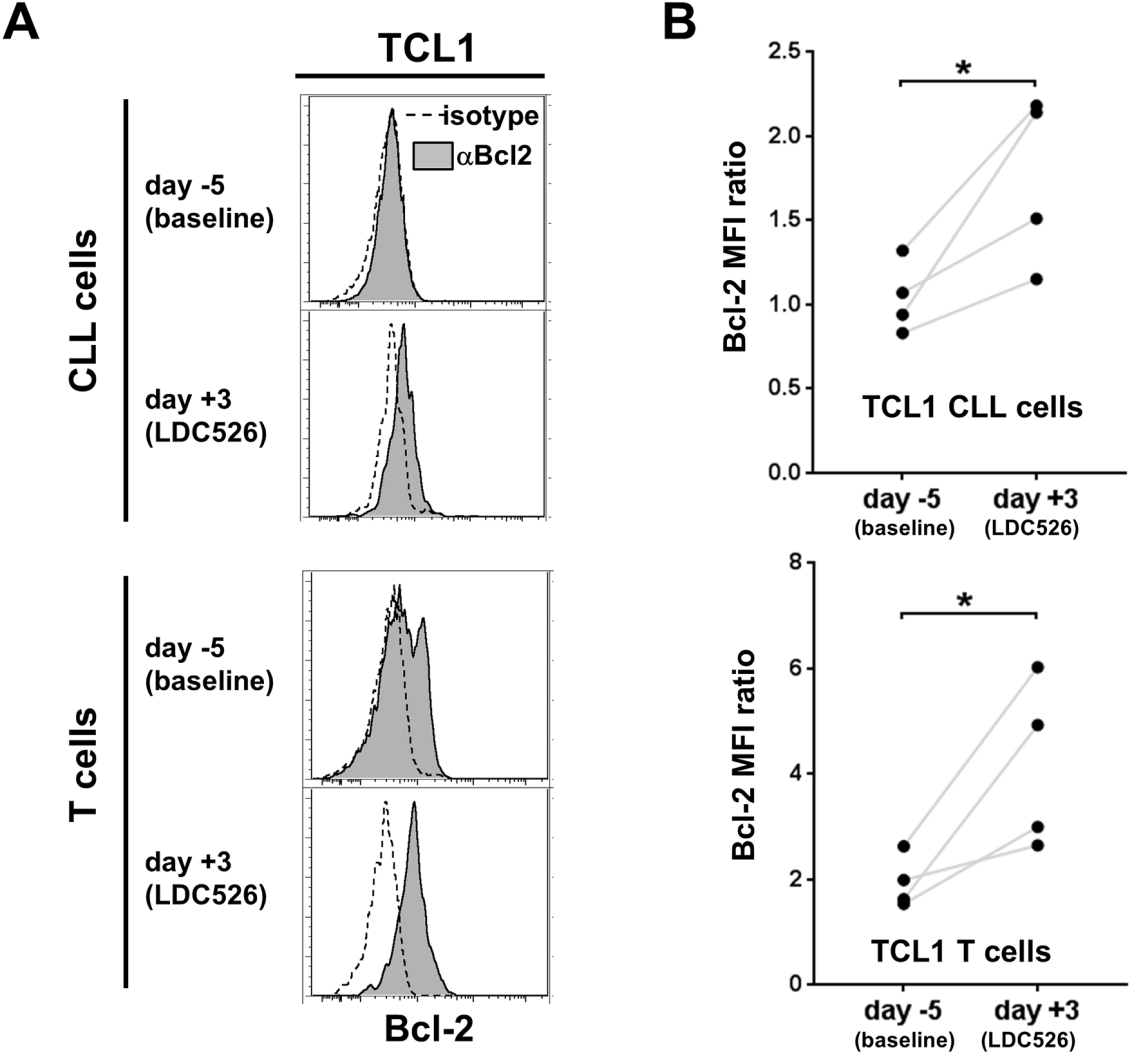
Residual CLL cells and T cells post LDC526 treatment express high levels of BCL-2 **(A)** Representative histograms of intracellular anti-Bcl-2 and corresponding isotype control staining of TCL1 CLL cells (CD5+CD19+) and T cells (CD5+CD19-). **(B)** Graphs comparing the CLL and T cell intracellular Bcl-2 expression of individual mice on days -5 and +3. The Bcl-2 expression was quantified as median fluorescence intensity ratio (MFI ratio: MFI anti-Bcl-2/MFI isotype control antibody). ^*^ p<0.05.

Inhibition of CDK9 activity prevents RNA transcriptional elongation and deprives cells from the short-lived antiapoptotic protein MCL-1 leading to apoptosis [37]. Given the redundancy between the antiapoptotic proteins MCL-1 and BCL-2 we intended to study the effect of LDC526 on longitudinal cellular BCL-2 levels. We measured Bcl-2 protein levels in TCL1 lymphocyte subsets by flow cytometric analysis on days -5 and +3 (Figure 7A) of the experimental set-up outlined in Figure 5A. As mentioned before, peripheral blood TCL1 CLL cells on day +3 were diminished to 6.4% of baseline levels by LDC526 treatment (Figure 6C). Remarkably, residual CLL cells exhibited significantly increased intracellular Bcl-2 levels after LDC526 exposure. Moreover, T cells that were decreased in numbers by LDC526 treatment also displayed significantly higher Bcl-2 levels compared to levels measured before LDC526 application (Figure 7B). We speculate that inherent higher cellular Bcl-2 levels of a subset of TCL1 CLL and T cells might have protected these cells from apoptosis by LDC526-mediated MCL-1 loss. Alternatively, LDC526 treatment might have induced upregulation of Bcl-2 by a so far unknown mechanism in residual cells.

## DISCUSSION

An increasing number of studies support the notion that pan-CDK inhibitors such as flavopiridol (Alvocidip) exert their primary anti-leukemic CLL activity by interfering with transcription via CDK9 inhibition rather than inhibiting the cell cycle [18, 38, 39]. Because pan-CDK inhibitors were associated with a narrow therapeutic window, it is anticipated that specific CDK9 inhibition would selectively inhibit transcription and not affect cell cycle progression and might therefore exhibit a more favorable safety profile. Compared to other CDK inhibitors such as flavopiridol and SNS-032 our novel CDK9 inhibitor LDC526 exhibited improved selectivity for CDK9. Meanwhile, LDC526 was optimized further in terms of pharmacological parameters resulting in BAY1143572 [27], which has been studied in phase I trials in patients with acute leukemia and solid tumors / lymphomas (ClinicalTrials.gov, Identifier NCT02345382 and NCT01938638, respectively).

Here, we first studied the anti-CLL activity of LDC526 *in vitro* to provide the basis for subsequent *in vivo* studies carried out in the CLL NSG xenograft and the TCL1 transgenic CLL mouse model. Recently, other groups described other CDK9 inhibitors with improved CDK9 selectivity profiles such as CDKI-73 and LY2857585 [40–42]. The biochemical LY2857585 selectivity for CDK9 versus for example CDK7 was lower (22-fold;[42]) than the LDC526 CDK9 selectivity versus CDK7 (52-fold) described here. The degree of *ex vivo* CLL cell killing by LDC526 in the sub-micromolar range was comparable to the CLL cell killing achieved by LY2857585 and CDKI-73 [40–42].

Next, we moved on to *in vivo* testing of LDC526 in two independent mouse models of CLL. To our knowledge, there are only two other studies using the complementary CLL NSG xenograft and the TCL1 transgenic CLL mouse in preclinical drug testing [43, 44]. This is the first study investigating the impact of a specific CDK9 inhibitor in CLL *in vivo* models. The use of *in vivo* models has the advantage of providing evidence whether the interaction of CLL and stroma influences the response to CDK9 inhibition. This is of particular importance since the interaction of CLL with stroma *in vivo* leads to increased CDK9 activity and elevated MCL-1 expression in CLL cells [45, 46]. Here, we demonstrated that LDC526 has dose-dependent *in vivo* anti-CLL activity in the NSG xenograft model. The insufficient LDC526 anti-CLL activity of the 50 mg/kg dose might well be explained by prevailing high stroma-induced expression levels of anti-apoptotic proteins by CLL cells in the NSG spleen, which were not present in the *in vitro* setting.

In contrast to human CLL cells, the lower LDC526 50 mg/kg dose was already capable of inducing significant anti-human T cell activity within the NSG spleen. Altogether, the LDC526 anti-human T cell activity appeared more pronounced than the anti-CLL activity in the NSG spleen environment. This might be due to fact that activated T cells involved in an alloreactive (in the case of NSG, xenoreactive) response were shown to be especially sensitive to CDK9 inhibition [47, 48]. This is consistent with the upregulation of CDK9 upon T cell activation [49]. Since the proliferation of CLL cells was shown to be dependent on the presence of T cells in NSG spleens [29], the LDC526-mediated T cell reduction might have concomitantly contributed to the decreased splenic CLL cell numbers. However, it should be noted that residual T cells after 5 days of LDC526 therapy were primarily CD4+ T cells, which were shown to be indispensable for CLL proliferation in NSG spleens [29].

TCL1 mice mirror the course of human CLL as aged mice develop the clonal accumulation of CD5+CD19+ B cells in the blood and hematopoietic organs. Importantly, TCL1 mice display a response to CLL treatment analogous to human patients [32]. As disease onset and progression can be variable in TCL1 mice, we carried out a longitudinal study focusing on the course of peripheral blood CLL cell counts before and after LDC526 treatment. In parallel, we determined standard blood counts. In contrast to mean WBC dropping 12-fold in TCL1 mice after LDC526 treatment, mean platelet concentrations did not decrease below 50% of baseline levels. The decrease in platelet numbers was expected as CDK9/pTEFb is involved in megakaryocyte maturation [50]. Possibly the platelet drop turned out relatively subtle because other CDKs involved in megakaryocyte differentiation and functions were not targeted by LDC526. Treating TCL1 mice with LDC526 resulted in a reduction of blood CLL cells to 6.4% of baseline values, which is a striking response for a two-day oral therapy. Comparability between the LDC526 CLL responses in TCL1 blood versus the NSG spleen is limited. Besides the species disparities between CLL cells studied, the difference observed in the magnitude of CLL response obtained in blood (TCL1) versus spleen (NSG) could be explained by the micro-environmental pro-survival signals CLL cells are exposed to in the NSG spleen which are not provided in the TCL1 circulation. In the NSG spleen pro-survival and proliferation signals are presumably delivered to human CLL cells within patient-derived follicular structures that were described to be surrounded by human T cells [29].

Our study demonstrated *in vivo* cytotoxicity of non-malignant T cells towards specific CDK9 inhibition in the murine as well in the human CLL NSG system. Interestingly, TCL1 T cells exhibited a higher degree of LDC526 sensitivity than T cells of wild-type control mice without CLL. Others and we have shown skewing of the T cell compartment in CLL patients and in TCL1 mice towards an effector memory phenotype [51–53]. This might explain the higher LDC526 sensitivity of TCL1 CLL T cells compared to controls since the effector memory T cell subpopulations were shown to upregulate CDK9 and Cyclin T1 [49]. Moreover, LDC526 targeting of stromal non-malignant T cells exerting a CLL-supportive impact might have indirectly contributed to the LDC526 *in vivo* anti-CLL effect. On the other hand CD8+ T cells even though mainly dysfunctional in the CLL setting [51, 52, 54] might still exert some anti-tumoral effects that would be depleted by LDC526 therapy. Furthermore, an increased rate of infectious complications due to decreased lymphocyte counts would be expected to emerge under LDC526 treatment.

Recently, direct *in vivo* cytotoxicity of the selective CDK9 inhibitor BAY1143572 against malignant T cells (adult T cell leukemia/lymphoma, ATL) was shown. BAY1143572 also exerted a cytotoxic effect against normal CD4+ T cells *in vitro*, which was not as prominent as the effect against ATL cells [27].

Remarkably, we demonstrated that LDC526 has a cytotoxic effect on non-malignant B cells *in vivo*. This effect involved normal B cells of diseased CLL TCL1 mice as well as circulating and splenic B cells of wild-type mice. LDC526 induced MCL-1 loss is likely responsible for the depletion of B cells since MCL-1 is required for the survival of most normal B cell subsets [55]. However, the LDC526-induced B cell loss was not complete since circulating B cell numbers were rising 10 days post the cessation of LDC526 treatment and splenic B cell numbers of LDC526-treated wild-type mice corresponded to 31% of splenic B cell numbers of untreated mice.

We demonstrated in TCL1 CLL mice that residual CLL cells post LDC526 treatment exhibited higher Bcl-2 levels than CLL cells before treatment. Interestingly, others described a similar upregulation of BCL-2 in CLL cells post CDK inhibitor exposure *in vitro* (24 hours) and patient treatment (100 mg/m^2^) with the pan-CDK inhibitor SNS-032 [18, 21]. The mechanism of how CDK9 inhibition might lead to higher cellular BCL-2 levels remains elusive. However, as transcription is suppressed by CDK9 inhibition upregulated BCL-2 mRNA expression appears an unlikely cause. Instead, CLL cells with *a priori* higher BCL-2 levels might exhibit a selective advantage to survive MCL-1 depletion by CDK9 inhibitor treatment. Remarkably, higher BCL-2 levels within lymphoma cells were shown to correlate with an increased sensitivity to venetoclax treatment [56]. Therefore, we think high BCL-2 levels in residual CLL cells after CDK9 inhibitor therapy represent a rationale for combining CDK9 inhibitor treatment with a BCL-2 inhibitor such as venetoclax to achieve deeper CLL remissions or potentially cure. In line with this approach, CDK inhibition with flavopiridol or dinaciclib synergized with venetoclax to induce apoptosis of lymphoma cell lines *in vitro* [57, 58].

In conclusion, we describe the novel specific CDK9 inhibitor LDC526 and demonstrate effective LDC526 anti-leukemic activity in two independent preclinical CLL mouse models. Residual CLL cells after LDC526 treatment were characterized by higher BCL-2 levels. Therefore, specific CDK9 inhibitors are primary candidates for combination therapy with BCL-2-inhibitors to further deepen and prolong CLL treatment responses.

## MATERIALS AND METHODS

### Cells and cell culture

The MEC-1 cell line was obtained from the DSMZ (Braunschweig, Germany) and was cultured in RPMI (10% fetal bovine serum). A LDC526 stock solution was prepared with DMSO, diluted and added to the cultures to reach the specified concentrations. Samples (percentage of CD19+CD5+ CLL cells >90%) of n=37 CLL patients (Table 1) and n=6 healthy normal donor controls were used for experiments after obtaining informed consent according to institutional guidelines, approved by the Ethics Commission of the University of Essen-Duisburg. Peripheral blood mononuclear cells (PBMC) were isolated using Lymphoprep (STEMCELL Technologies, Cologne, Germany) density gradient centrifugation and were used directly for NSG transplantation or *in vitro* experiments. Untouched normal donor B cells were isolated with a B cell isolation kit (Miltenyi, Bergisch Gladbach, Germany) according to the manufacturer’s instructions. For *in vitro* experiments, cells were maintained in RPMI supplemented with 10% fetal bovine serum, penicillin and streptomycin.

### Mice

All mice were bred and housed at the University Hospital Essen animal care facility. TCL1 mice [31] were back-crossed more than 6 generations into the C57BL/6 background and were genotyped as previously described [51]. Age-matched TCL1 wild-type littermates served as controls. Peripheral blood counts were determined with the VetABC analyzer (scil, Viernheim, Germany). Blood smears were Wright-Giemsa stained. For the human CLL xenograft experiments eight to 14-week-old NOD.Cg-Prkdc^scid^ Il2rg^tm1Wjl^/SzJ (NOD/scid/γc^null^; NSG) mice were sublethally irradiated (3.25 Gy) 24 hours before transplantation. Freshly isolated 1.0 x 10^8^ PBMC suspended in 0.2 ml of RPMI were intravenously transplanted as previously described [30]. For oral administration, LDC526 was diluted in polyethelene glycol (PEG M_n_ 400, Sigma-Aldrich) and administerd by gavage. Animal experiments were performed in accordance with institutional guidelines approved by the Animal Care Committee of the University Hospital Essen.

### Inhibitors

LDC526 [(3-((4-(4-fluoro-2-methoxyphenyl)-1,3,5-triazin-2-yl)amino)phenyl)methane-sulfonamide, molecular weight 389,41 g/mol, Figure 1A] was provided as powder (LDC526 synthesis, supplementary methods). For oral gavage administration LDC526 was diluted in polyethelene glycol (PEG M_n_ 400, Sigma-Aldrich, Munich, Germany) followed by sonication for 3 x 5 minutes with intermediate mixing. The CDK inhibitors SNS-032, R-547 (Selleckchem, Munich, Germany) and Flavopiridol (Sigma-Aldrich) were diluted in DMSO.

### *In vitro* enzymatic kinase assay for CDKs

IC50 values for CDK inhibitors were determined using the fluorescence resonance energy transfer (FRET)-based LANCE Ultra KinaSelect Ser/Thr kit (Perkin Elmer). Kinase activity and inhibition were measured according to the manufacturer’s instructions and as previously described [59]. Briefly, a specific ULight MBP peptide substrate (50 nM final concentration) was allowed to get phosphorylated by a CDK-cyclin pair in enzymatic buffer (50 mM Hepes pH 7.5, 10 mM MgCl2, 1 mM EGTA, 2 mM dithiothreitol) containing ATP at the concentration of the Km values of the individual kinases for 1 hour at room temperature. Subsequently, phosporylation was detected by addition of specific europium (Eu)-labeled anti-phospho-antibodies (2 nM), which upon binding to the phospho-peptide give rise to a FRET signal. FRET signals were recorded in a time-resolved manner in a Perkin Elmer EnVision reader. Purified cyclin-kinase pairs were obtained from the following suppliers: Carna Biosciences (CDK1-Cyclin B1, CDK6-Cyclin D3, CDK7-Cyclin H-MAT1), ProQinase (CDK2-Cyclin A) and Invitrogen (CDK9-Cyclin T1). Broad selectivity kinase *in vitro* profiling with LDC526 was carried out using n=219 recombinant kinases (Millipore/Merck, Darmstadt, Germany).

### Cell preparation and flow cytometry

Apoptosis and cell death were determined by using Annexin V (BD Biosciences, Heidelberg, Germany) and 4′,6-Diamidin-2-phenylindol (DAPI, Sigma-Aldrich) staining. Erythrocytes of murine peripheral blood samples were lysed with NH_4_Cl hypotonic solution. Single cell suspensions of murine spleens were prepared as previously described [60]. Murine cells were stained with antibodies directed against murine CD3 (clone 145-2C11), CD5 (53-7.3) and CD19 (1D3), respectively. For the detection of human cells in NSG spleens antibodies against human CD3 (clone SK7), CD4 (SK3), CD5 (L17F12), CD8 (SK1), CD19 (J3-119 or SJ25C1) and CD45 (2D1) were used (purchased from BD Bioscience, Biolegend and Beckman Coulter). Flow cytometric analysis was carried out on a LSRII flow cytometer (BD Biosciences, Heidelberg, Germany). Flow cytometric data were analyzed with BD FACSDiva or FlowJo software (FLOWJO, LLC, Ashland, OR, USA). Intracellular staining for MCL-1 and BCL-2 (anti-MCL-1 [clone D2W9E, Cell Signaling Technology, Leiden, The Netherlands] and matched isotype control [clone DA1E, Cell Signaling Technology]; anti-BCL-2 [clone Bcl-2/100, BD Bioscience] and matched isotype control [clone MOPC-21, BD Bioscience]) within MEC-1 and primary CLL cells was performed with the FoxP3 / Transcription factor staining buffer set (eBioscience/Thermo Fisher Scientific, Dreieich, Germany) according to the manufacturer’s instructions. Before permeabilization and antibody incubation cells were stained with a fixable viability dye (eFluor 450, eBioscience/Thermo Fisher Scientific). Before adding the anti-MCL-1 and anti-BCL-2 antibodies permeabilized cells were blocked with 2% mouse serum and 2% rabbit serum (Sigma-Aldrich) in permeabilization buffer. Intracellullar murine lymphocyte staining for Bcl-2 was carried out with the BD Cytofix/Cytoperm kit according to the manufacturer’s instructions. A Hamster Anti-mouse Bcl-2 (clone 3F119, BD Biosciences) and a corresponding isotype control (clone A19-3) were used. Dead cells and doublets were excluded by and FSC/SSC gating and positive DAPI or eFluor 450 viability dye staining, respectively. To assess the impact of LDC526 on the cell-cycle status of MEC-1 cells we used the BrdU Flow Kit according to the manufacturer’s protocol (BD Bioscience). BrdU was added to the media 30 minutes before commencing the analysis.

### Imaging

Macroscopic spleen images were taken with a Canon IXUS 1100 HS digital camera (Canon, Krefeld, Germany). Images of stained blood smears of LDC526 treated mice were taken through the 10x and 50x objective lenses of an Axioskope microscope (Zeiss, Jena, Germany).

### Statistical analyses

Data were analyzed with GraphPad Prism 7 software. Differences were assessed by unpaired or paired Student t tests if not indicated otherwise. Two-sided probabilities of less than 0.05 were considered significant. Nonlinear regression sigmoidal dose-response curve fit was used to determine the IC_50_ values and to generate graphs. All data are displayed as mean±SEM.

## SUPPLEMENTARY MATERIALS FIGURES



## Abbreviations

BCL-2: B-cell lymphoma/leukemia 2
BTK: Bruton's tyrosine kinase
CDK9: cyclin-dependent kinase 9
CLL: chronic lymphocytic leukemia
CTD: carboxyterminal domain
DAPI: 4′,6-Diamidin-2-phenylindol
FISH: fluorescent *in situ* hybridization
FRET: fluorescence resonance energy transfer
MCL-1: Myeloid cell leukemia 1 (MCL-1)
NSG: NOD/scid/γc^null^
PBMC: peripheral blood mononuclear cells
PI3Kδ: phosphatidylinositol-3-kinase delta
RNAPII: RNA Polymerase II
pTEFb: transcription elongation factor b
WBC: White blood cell count
