# Supplementary material for: Potent anti-leukemic activity of a specific cyclin-dependent kinase 9 inhibitor in mouse models of chronic lymphocytic leukemia
Source: Oncotarget. 2018 May 29;9(41):26353–69. doi: 10.18632/oncotarget.25293 (PMC5995184; doi:10.18632/oncotarget.25293)
Supplement: Supplementary file 1 [file oncotarget-09-26353-s001.pdf]

# Potent anti-leukemic activity of a specific cyclin-dependent kinase 9 inhibitor in mouse models of chronic lymphocytic leukemia

## SUPPLEMENTARY MATERIALS

### Synthesis of LDC526

#### 3-[(4-Chloro-1,3,5-triazin-2-yl)amino] benzenemethanesulfonamide

To a solution of 2,4-dichloro-1,3,5-triazine (900 mg, 6.0 mmol) in dry tetrahydrofuran/isopropanol (1/1 mixture, 8 mL) at -20°C under $N_2$ atmosphere was added a cooled solution (-20°C) of (3-aminophenyl)-methanesulfonamide (1117 mg, 6.0 mmol) and DIPEA (2.07 mL, 12 mmol) in dry tetrahydrofuran/isopropanol (1/1 mixture, 8 mL). The reaction mixture was stirred for 2 hours at -10°C. The mixture was concentrated under reduced pressure and the crude product was dried in vacuo for 15 hours. The white solid was used in the next step without further purification. $^1$H NMR (400 MHz, [$D_6$]DMSO, 300 K): δ = 10.83 (s, 1H), 9.75 (br. s, 1H), 8.64 (s, 1H), 7.66 (d, 1H), 7.36 (d, 1H), 7.15 (d, 1H), 6.89 (s, 2H), 4.26 (s, 2H). MS (ES): m/z $[M+H]^+$ calcd. for $C_{10}H_{10}ClN_5O_2S$: 300, found: 300.

#### 1-(3-{[4-(4-Fluoro-2-methoxyphenyl)-1,3,5-triazin-2-yl]amino}phenyl)-methanesulfonamide (LDC526)

A mixture of 3-[(4-chloro-1,3,5-triazin-2-yl)amino] benzenemethanesulfonamide (1767 mg, 5.91 mmol), 4-fluoro-2-methoxyphenylboronic acid (1504 mg, 8.85 mmol) and tripotassium phosphate (2505 mg, 11.82 mmol) in dioxane/water (10/1 mixture, 66 mL) was degassed with a stream of $N_2$ for 15 minutes. Pd(dppf)$Cl_2 \cdot CH_2Cl_2$ (489 mg, 0.6 mmol) was added and the reaction mixture was heated for 90 min at 145°C in a microwave oven. The mixture was diluted with ethyl acetate and washed with saturated aqueous sodium bicarbonate solution. The organic phase was dried ($Na_2SO_4$), filtered and concentrated. The crude was purified by flash chromatography (ethyl acetate/methanol, 100:0 to 5:1). Finally, precipitation from ethyl acetate yielded the desired product (129 mg, 0.33 mmol, 6%) as a white solid. $^1$H NMR (400 MHz, [$D_6$]DMSO, 300 K): δ = 10.32 (s, 1H), 8.78 (s, 1H), 7.89 (br. s, 2H), 7.72 (s, 1H), 7.34 (t, J = 7.9 Hz, 1H), 7.07 (m, 2H), 6.91 – 6.82 (m, 3H), 4.22 (s, 2H), 3.87 (s, 3H). MS (ES): m/z $[M+H]^+$ calcd. for $C_{17}H_{16}FN_5O_3S$: 390, found: 390.

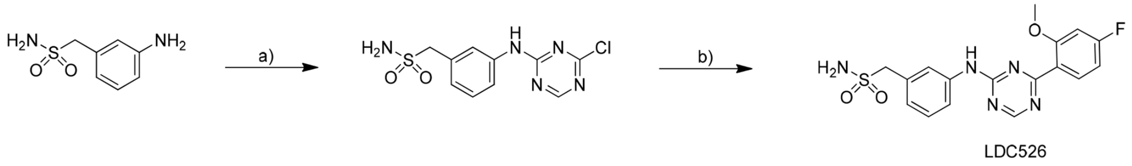


**Scheme of LDC526 synthesis.** *Reagents and conditions*: **(a)** 2,4-dichloro-1,3,5-triazine, DIPEA, THF/*i*PrOH (1:1), –20 °C to –10 °C, 2 h; **(b)** 4-fluoro-2-methoxyphenylboronic acid, Pd(dppf)$Cl_2 \cdot CH_2Cl_2$, $K_3PO_4$, dioxane/water (10:1), 140–145 °C, microwave oven, 90min.

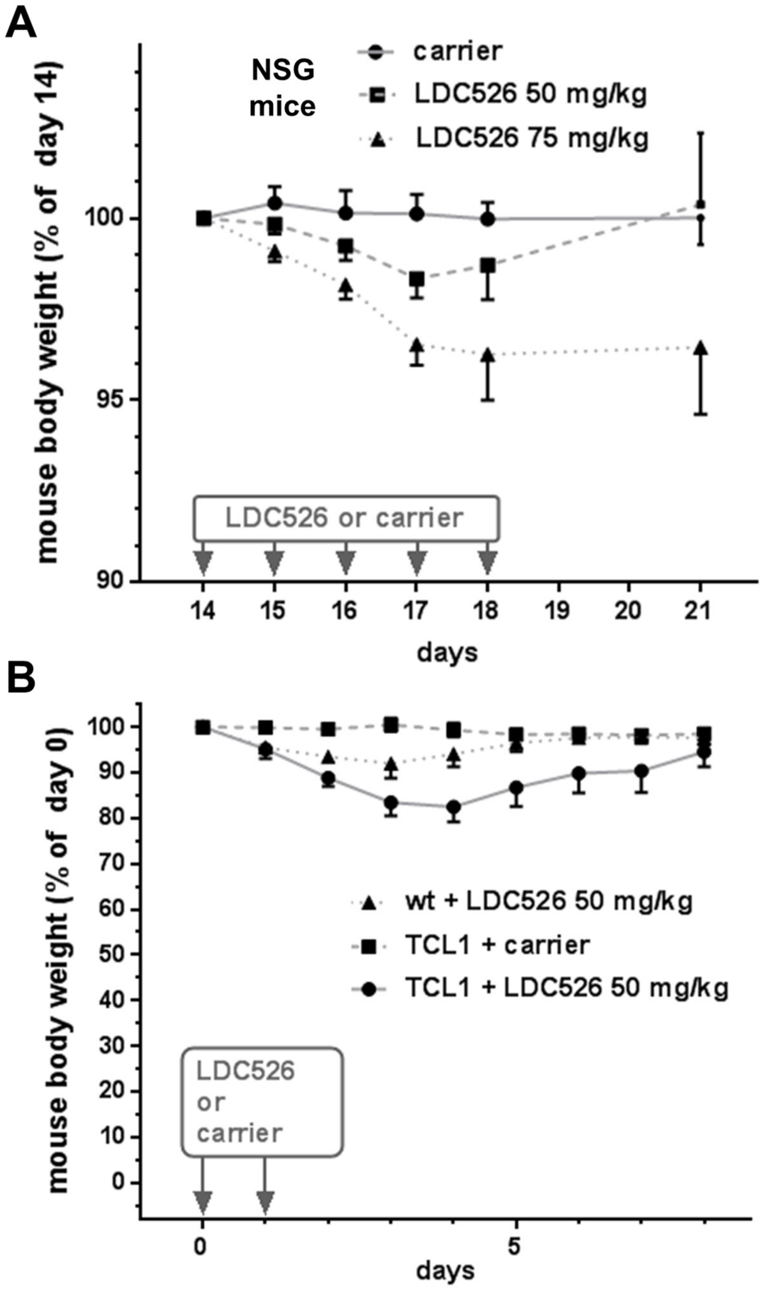


**Supplementary Figure 1: Body weight and LDC526 drug schedule of CLL-transplanted NSG and TCL1 transgenic mice. (A)** Different numbers of NSG mice per group and day (experimental set-up Figure 3A) were weighed (Carrier control: day (d) 14-17, mice n=21; d18, n=10; d21, n=7/LDC526 50 mg/kg: d14-17, n=18; d18, n=8 ; d21, n=6/LDC526 75 mg/kg: d14-17, n=18; d18, n=7; d21, n=4). **(B)** The weight of mice (experimental set-up Figure 5A) was determined on the indicated days. Percentages of weight relate to the initial weight (day 0) just before the first carrier control or LDC526 doses were administered. On day 7 one LDC526-treated TCL1 was in reduced general condition and had to be euthanized. Data points with error bars represent means+/-SEM.

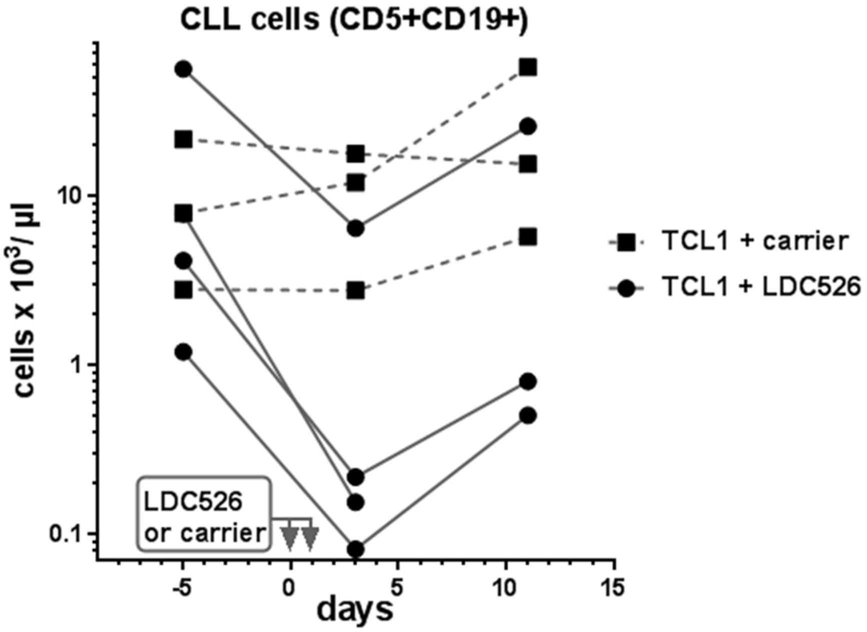


**Supplementary Figure 2: Dot plot graph displaying peripheral blood CLL counts of individual TCL1 mice.** Same data as shown in Figure 6B as mean±SEM.

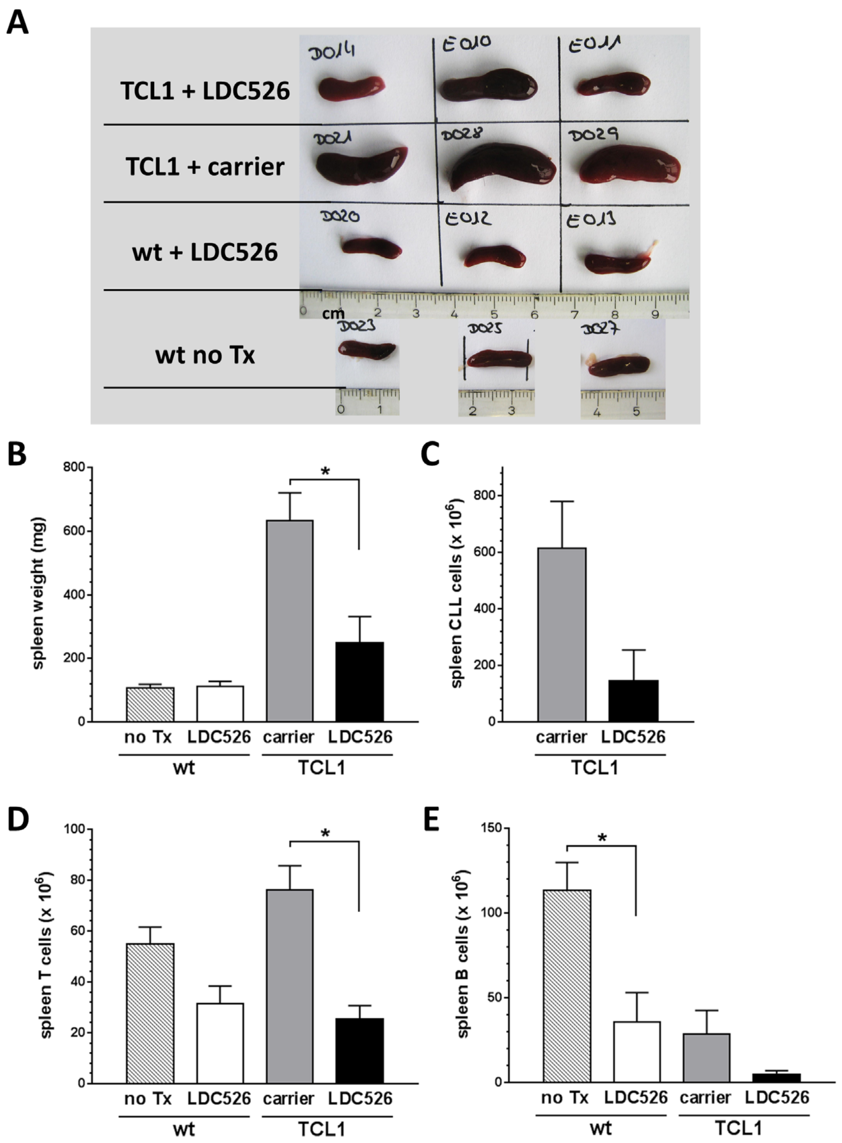


**Supplementary Figure 3: Analysis of TCL1 spleens 10 days after LDC526 treatment.** **(A)** Macroscopic spleen images. Analysis was carried out on day 11 (experimental scheme as outlined in Figure 5A). Age-matched wild-type littermate control mice neither receiving LDC526 nor carrier treament (wt no Tx) were included in the analysis as an additional control group. Quantification of spleen **(B)** weights, **(C)** CLL (CD5+CD19+) cell numbers, **(D)** T cells (CD5+CD19-CD3+) numbers and **(E)** non-malignant B cell (CD5-CD19+CD3-) numbers. Tx, treatment. $^{*}$ $p<0.05$.
